# Supplementary material for: Polyketide synthase gene clusters in the ericoid mycorrhizal fungus Oidiodendron maius: genome-wide prediction, expression profiling, and functional characterization of knockout mutants
Source: Front Plant Sci. 2026 Sep 16;17:1924430. doi: 10.3389/fpls.2026.1924430 (PMC13623903; doi:10.3389/fpls.2026.1924430)
Supplement: Supplementary file 1 [file SupplementaryFile1.zip › Supplementary Materials/Supplementary Folder 1.zip!/Oidma1_AssemblyScaffolds_Repeatmasked/knownclusterblast/cluster48/jgi.p_Oidma1_45847_mibig_hits.html]

| MIBiG Protein | Description | MIBiG Cluster | MiBiG Product | % ID | % Coverage | BLAST Score | E-value |
| --- | --- | --- | --- | --- | --- | --- | --- |
| ACS68556.1 | major\_facilitator\_superfamily\_protein | BGC0001026 | NG-391\_biosynthetic\_gene\_cluster | 40 | 96.1904761905 | 416 | 3.4e-116 |
| BAE56594.1 |  | BGC0001123 | Aspirochlorine\_biosynthetic\_gene\_cluster | 41 | 99.8095238095 | 405 | 7.9e-113 |
| ADY16699.1 | TqaJ | BGC0001142 | Tryptoquialanine\_biosynthetic\_gene\_cluster | 28 | 90.2857142857 | 234 | 3.2e-61 |
| AFU65888.1 | DacR2 | BGC0000216 | Dactylocycline\_biosynthetic\_gene\_cluster | 31 | 93.5238095238 | 207 | 4.1e-53 |
| AAQ08935.1 | putative\_membrane\_transporter | BGC0000224 | Fredericamycin\_biosynthetic\_gene\_cluster | 27 | 94.2857142857 | 198 | 1.5e-50 |
| ADU85991.1 | putative\_membrane\_transport\_protein | BGC0000165 | Tiacumicin\_B\_biosynthetic\_gene\_cluster | 32 | 76.0 | 188 | 2.6e-47 |
| CCE28984.1 | probable\_DHA14-like\_major\_facilitator;\_ABC\_transporter | BGC0001365 | Clapurines\_biosynthetic\_gene\_cluster | 27 | 81.9047619048 | 180 | 4.1e-45 |
| AGC95323.1 | CurE | BGC0000045 | Dehydrocurvularin\_biosynthetic\_gene\_cluster | 28 | 77.1428571429 | 173 | 5.1e-43 |
| ADG86335.1 | transporter | BGC0000190 | A-74528\_biosynthetic\_gene\_cluster | 26 | 94.8571428571 | 171 | 1.9e-42 |
| ACP19369.1 | SaqJ1 | BGC0000267 | Saquayamycin\_Z\_/\_galtamycin\_B\_biosynthetic\_gene\_cluster | 30 | 78.8571428571 | 170 | 5.6e-42 |
| ACD39765.1 | major\_facilitator\_superfamily\_transporter | BGC0000077 | Hypothemycin\_biosynthetic\_gene\_cluster | 28 | 76.5714285714 | 168 | 2.1e-41 |
| ACD39756.1 | major\_facilitator\_superfamily\_transporter | BGC0000076 | Hypothemycin\_biosynthetic\_gene\_cluster | 28 | 76.5714285714 | 168 | 2.1e-41 |
| CAA09636.1 | putative\_transmembrane\_protein | BGC0000227 | Granaticin\_biosynthetic\_gene\_cluster | 30 | 77.5238095238 | 161 | 2e-39 |
| ADM34144.1 | efflux\_pump | BGC0001084 | Notoamide\_/\_stephacidin\_biosynthetic\_gene\_cluster | 27 | 78.8571428571 | 161 | 2e-39 |
| ABC87523.1 | putative\_drug\_efflux\_transporter | BGC0001011 | Meridamycin\_biosynthetic\_gene\_cluster | 28 | 78.4761904762 | 143 | 9.6e-34 |
| ABX71121.1 | Lct38 | BGC0000238 | Lactonamycin\_biosynthetic\_gene\_cluster | 28 | 90.0952380952 | 142 | 1.6e-33 |
| ABX71155.1 | Lcz38 | BGC0000237 | Lactonamycin\_Z\_biosynthetic\_gene\_cluster | 27 | 85.1428571429 | 128 | 1.9e-29 |
| CAE51185.1 | RemN\_protein | BGC0000264 | Resistomycin\_biosynthetic\_gene\_cluster | 28 | 78.4761904762 | 125 | 1.6e-28 |
| AEK75506.1 | EmrB/QacA\_drug\_resistance\_transporter | BGC0000001 | Abyssomicin\_biosynthetic\_gene\_cluster | 25 | 85.1428571429 | 124 | 3.5e-28 |
| AAQ08939.1 | putative\_membrane\_transporter | BGC0000224 | Fredericamycin\_biosynthetic\_gene\_cluster | 29 | 74.0952380952 | 120 | 5.1e-27 |
| ACB37757.1 | putative\_multidrug\_export\_protein | BGC0000162 | Tetrocarcin\_A\_biosynthetic\_gene\_cluster | 26 | 83.8095238095 | 120 | 6.6e-27 |
| AGC09506.1 | LobA4 | BGC0001183 | Lobophorin\_biosynthetic\_gene\_cluster | 25 | 79.2380952381 | 119 | 8.7e-27 |
| AGI99475.1 | efflux\_permease | BGC0001004 | Lobophorin\_biosynthetic\_gene\_cluster | 25 | 79.2380952381 | 118 | 2.5e-26 |
| AAM77999.1 | transmembrane\_transporter | BGC0000112 | Neocarzinostatin\_biosynthetic\_gene\_cluster | 29 | 78.8571428571 | 115 | 2.1e-25 |
| ABP54623.1 | drug\_resistance\_transporter,\_EmrB/QacA\_subfamily | BGC0000241 | Lomaiviticin\_biosynthetic\_gene\_cluster | 27 | 75.619047619 | 106 | 1.3e-22 |
| ADD83011.1 | PtnP4 | BGC0001156 | Platencin\_biosynthetic\_gene\_cluster | 24 | 80.0 | 103 | 6.4e-22 |
| ACS13711.1 | PtmP4 | BGC0001140 | Platensimycin\_/\_platencin\_biosynthetic\_gene\_cluster | 24 | 90.8571428571 | 102 | 1.4e-21 |
| ACN38360.1 | putative\_sisomicin\_exporter | BGC0000714 | Sisomicin\_biosynthetic\_gene\_cluster | 26 | 79.619047619 | 99 | 1.6e-20 |
| AHZ61835.1 | Lom1 | BGC0000240 | Lomaiviticin\_biosynthetic\_gene\_cluster | 26 | 75.8095238095 | 90 | 7.4e-18 |
| AEA60653.1 | transporter | BGC0000892 | Caryoynencin\_biosynthetic\_gene\_cluster | 25 | 86.4761904762 | 87 | 3.7e-17 |
| CAQ64674.1 | putative\_MFS-type\_efflux\_protein | BGC0000087 | Lasalocid\_biosynthetic\_gene\_cluster | 25 | 90.8571428571 | 82 | 1.2e-15 |
| ACD39772.1 | major\_facilitator\_superfamily\_transporter | BGC0000134 | Radicicol\_biosynthetic\_gene\_cluster | 28 | 34.2857142857 | 77 | 6.5e-14 |
| KFH48706.1 | putative\_MFS-type\_transporter-like\_protein | BGC0000317 | Cephalosporin\_biosynthetic\_gene\_cluster | 23 | 31.2380952381 | 59 | 1.4e-08 |
